# Comparison of the efficacy of autogenous inactivated Porcine Reproductive and Respiratory Syndrome Virus (PRRSV) vaccines with that of commercial vaccines against homologous and heterologous challenges

**DOI:** 10.1186/1746-6148-8-182

**Published:** 2012-10-03

**Authors:** Marc F Geldhof, Merijn Vanhee, Wander Van Breedam, Jan Van Doorsselaere, Uladzimir U Karniychuk, Hans J Nauwynck

**Affiliations:** 1 Laboratory of Virology, Faculty of Veterinary Medicine, Ghent University, Salisburylaan 133, Merelbeke, 9820, Belgium; 2 Department of Health Care and Biotechnology, KATHO Catholic University College of South-West Flanders, Wilgenstraat 32, Roeselare, 8800, Belgium

## Abstract

**Background:**

The porcine reproductive and respiratory syndrome virus (PRRSV) is a rapidly evolving pathogen of swine. At present, there is a high demand for safe and more effective vaccines that can be adapted regularly to emerging virus variants. A recent study showed that, by the use of a controlled inactivation procedure, an experimental BEI-inactivated PRRSV vaccine can be developed that offers partial protection against homologous challenge with the prototype strain LV. At present, it is however not known if this vaccine can be adapted to currently circulating virus variants. In this study, two recent PRRSV field isolates (07 V063 and 08 V194) were used for BEI-inactivated vaccine production. The main objective of this study was to assess the efficacy of these experimental BEI-inactivated vaccines against homologous and heterologous challenge and to compare it with an experimental LV-based BEI-inactivated vaccine and commercial inactivated and attenuated vaccines. In addition, the induction of challenge virus-specific (neutralizing) antibodies by the different vaccines was assessed.

**Results:**

In a first experiment (challenge with 07 V063), vaccination with the experimental homologous (07 V063) inactivated vaccine shortened the viremic phase upon challenge with approximately 2 weeks compared to the mock-vaccinated control group. Vaccination with the commercial attenuated vaccines reduced the duration of viremia with approximately one week compared to the mock-vaccinated control group. In contrast, the experimental heterologous (LV) inactivated vaccine and the commercial inactivated vaccine did not influence viremia. Interestingly, both the homologous and the heterologous experimental inactivated vaccine induced 07 V063-specific neutralizing antibodies upon vaccination, while the commercial inactivated and attenuated vaccines failed to do so.

In the second experiment (challenge with 08 V194), use of the experimental homologous (08 V194) inactivated vaccine shortened viremia upon challenge with approximately 3 weeks compared to the mock-vaccinated control group. Similar results were obtained with the commercial attenuated vaccine. The experimental heterologous (07 V063 and LV) inactivated vaccines did not significantly alter viremia. In this experiment, 08 V194-specific neutralizing antibodies were induced by the experimental homologous and heterologous inactivated vaccines and a faster appearance post challenge was observed with the commercial attenuated vaccine.

**Conclusions:**

The experimental homologous inactivated vaccines significantly shortened viremia upon challenge. Despite the concerns regarding the efficacy of the commercial attenuated vaccines used on the farms where the field isolates were obtained, use of commercial attenuated vaccines clearly shortened the viremic phase upon challenge. In contrast, the experimental heterologous inactivated vaccines and the commercial inactivated vaccine had no or only a limited influence on viremia. The observation that homologous BEI-inactivated vaccines can provide a more or less standardized, predictable degree of protection against a specific virus variant suggests that such vaccines may prove useful in case virus variants emerge that escape the immunity induced by the attenuated vaccines.

## Background

Porcine reproductive and respiratory syndrome virus (PRRSV) infection is characterized by reproductive failure in sows, and is associated with respiratory problems in pigs of all ages [1-7]. With few exceptions, PRRSV is present in a majority of swine-producing countries around the world and gives rise to significant economic losses in the swine industry [8]. Based on genetic and antigenic analysis, two PRRSV genotypes are recognized: a European (EU) genotype (prototype: Lelystad virus, LV) [9] and a North American (NA) genotype (prototype: VR2332), which share about 55–70% nucleotide homology [10]. However, a high genetic variability has been demonstrated within both genotypes [10-13] and the genetic differences between virus variants are mirrored in different virulence, pathogenicity, immunogenicity, … A recent study by Diaz et al. (2012) showed that infection with different PRRSV strains leads to different virological and immunological outcomes and results in different degrees of homologous and heterologous protection [14]. Another study by Martinez-Lobo and coworkers (2011) reported that different PRRSV isolates differ in their susceptibility to antibody neutralization [15]. Evidently, the high variability of the virus represents a major hurdle for effective PRRSV prevention and control [16]. Since PRRSV poses a serious burden on the swine industry worldwide, the need for efficient control measures is high. A variety of PRRS eradication strategies have been described, including total depopulation/repopulation, partial depopulation, segregated early weaning, test and removal and herd closure. Also planned exposure to a farm-specific virus isolate is a common strategy in the United States and Canada [17]. This last approach is often performed without monitoring and is consequently unreliable in getting the targeted population homogeneously infected in a timely manner. While the above strategies can certainly be useful, it is also clear that efficient PRRSV vaccines are extremely valuable tools to minimize the clinical and economical impact of PRRSV infections. However, the commonly used vaccines, both attenuated and inactivated, are not without their problems. Although attenuated vaccines have the potential to protect animals against viremia, the degree of protection depends on various factors, including the homology between the vaccine virus and the circulating virus [18]. In addition, there are some safety concerns, as the vaccine virus may spread and revert to virulence [19-22]. The commercially available inactivated vaccines are generally safe to use, but do not provide sufficient protection [21,23,24]. In addition, the ability of PRRSV to subvert the host immune system further complicates these matters. At present, it is generally accepted that there is a need for new and safe vaccines that can protect against infection with those virus variants that escape immunity induced by the currently available commercial vaccines. In this context, the use of vaccine virus that is homologous to the PRRSV variants prevalent in the herd seems to be favourable [18]. Vanhee et al. (2009) demonstrated that, by use of a controlled inactivation procedure and a suitable adjuvant, an LV-based inactivated PRRSV vaccine can be developed that systematically induces an LV-specific virus-neutralizing (VN) antibody response upon 2 vaccinations in naïve piglets. Following homologous challenge of the vaccinated pigs with LV, animals developed an earlier and strongly elevated VN antibody response and a significant reduction of viremia was observed [25]. Currently however, it is unknown whether it is possible to achieve similar results for PRRSV isolates that are currently causing reproductive or respiratory disorders in the field. Two recent PRRSV isolates, from outbreaks in herds vaccinated with a registered vaccine, were used for autogenous inactivated vaccine development. The main objective of this study was to test the capacity of experimental inactivated autogenous PRRSV vaccines to protect naïve pigs against homologous PRRSV challenge and to compare the efficacy of these vaccines with that of experimental heterologous inactivated vaccines, the commercial vaccine used on the farms, and other commercial inactivated and attenuated vaccines.

## Results

### Vaccination experiment with PRRSV isolate 07 V063

#### Clinical examination

All animals remained in good health after they were vaccinated. No local or systemic vaccine side effects were noted throughout the trial period. No pigs died during the entire experimental period. Body temperatures fluctuated in all groups and statistically significant differences were not detected. Challenge with PRRSV isolate 07 V063 induced moderate fever (higher than 39.5°C, but not higher than 40.6°C) within 10 days post infection in 32 out of 36 inoculated pigs. The 4 remaining animals did not develop fever. By 11 days post challenge, fever had disappeared in all animals.

#### Viremia

**Figure 1:**
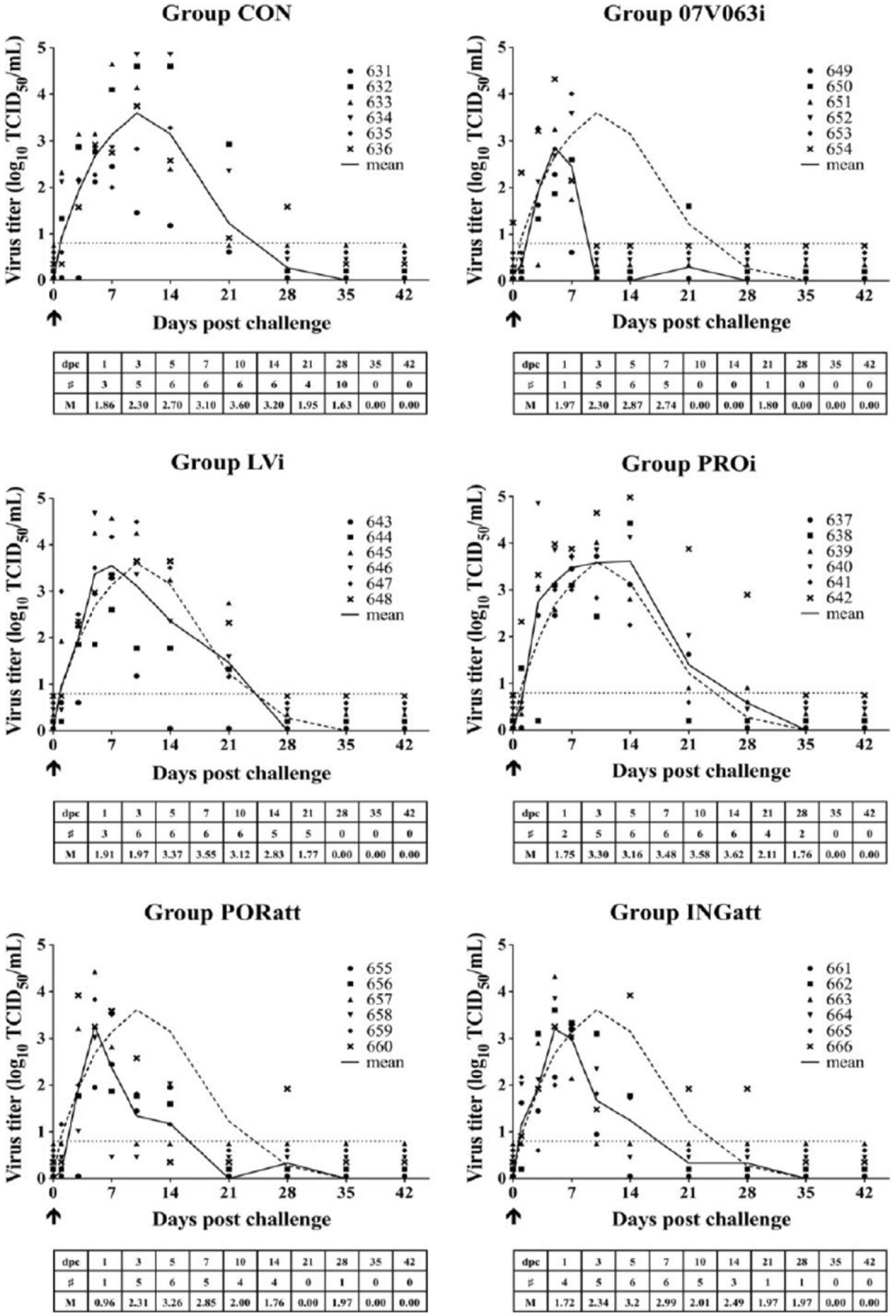
**Serum-virus titers after 07 V063 challenge for group CON (Mock-vaccinated control), 07 V063i (BEI-inactivated 07 V063), LVi (BEI-inactivated LV), PROi (Progressis®), PORatt (Porcilis® PRRS) and INGatt (Ingelvac® PRRS MLV).** Virus titers in serum (log_10_ TCID_50_/mL) were determined by virus titration on PAM, followed by immunoperoxidase staining for the PRRSV nucleocapsid protein. ↑ = challenge. Symbols represent individual animals and solid lines represent mean virus titers calculated on all animals present in each group. The dashed line indicates the mean titers for all animals in group CON. The dotted line marks the detection limit for virus titration. Mentioned in the table: # = the number of viremic animals in the different groups at different time points. M = mean virus titer of all viremic animals in the group at different time points.

Upon challenge, all animals became viremic. In the adjuvant control group (group CON), viremia was detected from day 1 after the challenge (3 pigs out of 6) and peaked around 10 days post challenge (dpc), with a mean virus titer of 3.6 log_10_ TCID_50_/mL. Viremia had cleared in all animals by 5 weeks post challenge (Figure 1, CON). In the binary ethyleneimine (BEI) inactivated 07 V063 group (group 07 V063i), all animals became viremic, but the peak viremia occurred earlier (day 5) and was lower (2.9 log_10_ TCID_50_/mL). From 10 dpc, virus was no longer detected in the serum of any of the animals, but one animal was again viremic at day 21 post challenge (Figure 1, 07 V063i). The mean viral titer in the serum was significantly reduced compared to the control group at days 10 and 14 (p < 0.05). In group 07 V063i, a significantly lower number of viremic piglets was observed compared to group CON on days 10 and 14 post challenge (p < 0.05). In the BEI-inactivated LV vaccinated group (group LVi), all animals became viremic after challenge, with a peak viremia of 3.6 log_10_ TCID_50_/mL on day 7. Four weeks post challenge, virus was not found anymore in the serum of any of the 6 pigs (Figure 1, LVi). The virus titers were not significantly lower than those of group CON. In the group vaccinated with Progressis® (group PROi), viremia was detected in all animals, with a peak of 3.6 log_10_ TCID_50_/mL around 10–14 dpc. The viremic phase showed a similar pattern as in group CON and virus titers were not significantly reduced. Viremia disappeared in all animals by 5 weeks after challenge (Figure 1, PROi). In the group vaccinated with a single shot of Porcilis® PRRS (group PORatt) and the group vaccinated with one dose of Ingelvac® PRRS (group INGatt), a partial reduction in viremia was seen. Viremia peaked at 5 dpc with average titers of 3.3 log_10_ TCID_50_/mL (group PORatt) and 3.2 log_10_ TCID_50_/mL (group INGatt) (Figure 1, PORatt and INGatt). No significant differences in mean virus titers were detected at any time-point between groups PORatt, INGatt and CON. Only at 5 weeks after challenge, all animals of these groups were consistently virus negative. Taking all data on viremia together, group 07 V063i was the only group that showed a significantly shortened viremia and a significant decrease in the number of viremic piglets compared to the mock-vaccinated control group.

#### 07 V063-specific antibodies

**Figure 2:**
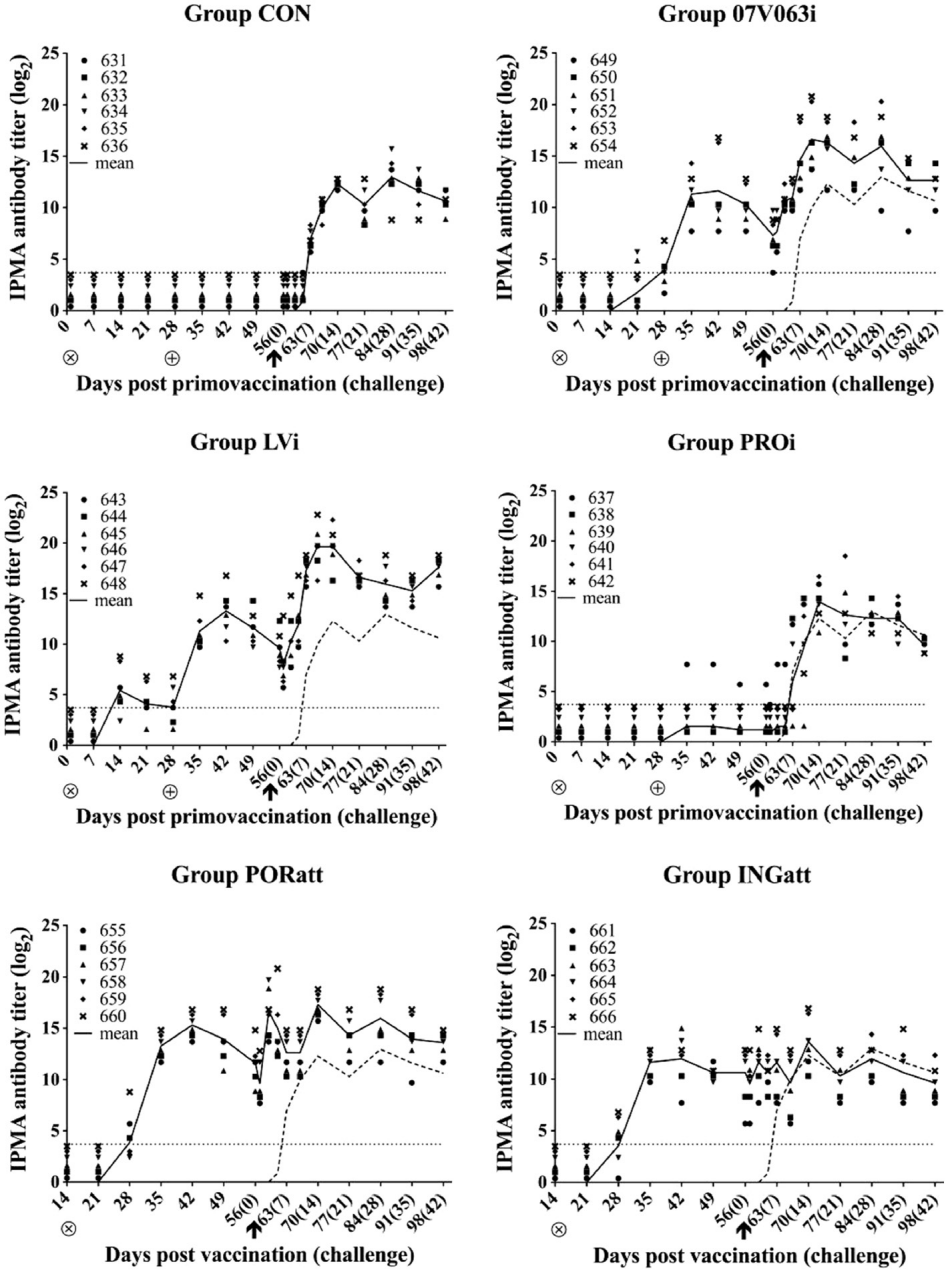
**07 V063-specific IPMA antibody titers (log**_**2**_**) after vaccination and 07 V063 challenge for group CON (Mock-vaccinated control), 07 V063i (BEI-inactivated 07 V063), LVi (BEI-inactivated LV), PROi (Progressis®), PORatt (Porcilis® PRRS) and INGatt (Ingelvac® PRRS MLV).** ⊗ = primo vaccination; ⊕ = booster vaccination; ↑ = challenge. Symbols represent individual animals and solid lines represent mean IPMA titers calculated on all animals present in each group. The dashed line indicates the mean titers for all animals in group CON. The dotted line marks the detection limit for the IPMA test.

In group CON, virus-specific antibodies were not detected before challenge (Figure 2, CON). At 7 dpc, antibodies could be detected in all animals of this control group. In 2 animals of group 07 V063i, antibodies could already be detected at 3 weeks after the primo vaccination. The remaining animals within this group became seropositive after booster vaccination. From the first week after booster vaccination until 10 days after challenge, antibody titers in this group remained significantly higher than in group CON (p < 0.05) (Figure 2, 07 V063i). In group LVi, virus-specific antibodies against 07 V063 were detected from 2 weeks after primo vaccination and all animals seroconverted after booster vaccination. Virus-specific antibody titers were significantly higher in group LVi compared to group CON from 7 days post booster vaccination until 21 dpc and at 42 dpc (p < 0.05) (Figure 2, LVi). In group PROi, one animal became seropositive at 1 week after booster vaccination, while the remaining animals did not show antibodies before challenge (Figure 2, PROi). Post challenge, the course of the antibody response of this group was similar as in group CON. In group PORatt, virus-specific antibodies were detected in 3 out of 6 pigs at 2 weeks after vaccination (Figure 2, PORatt). At 3 weeks post vaccination, all animals were seropositive. Virus-specific antibodies remained present during the entire experiment, and the antibody titers were significantly higher compared to group CON from 21 days post vaccination until 21 dpc (p < 0.05). In group INGatt, virus-specific antibodies were found in 3 out of 6 pigs at 2 weeks after vaccination (Figure 2, INGatt). One week later, all pigs had seroconverted and remained seropositive until the end of the experiment. Antibody titers were significantly higher compared to group CON from 21 days post vaccination till 5 dpc. Taken together, the courses of the IPMA antibody titers in all groups were similar to those described in other studies [24,25]. 

#### 07 V063-specific virus-neutralizing antibodies

**Figure 3:**
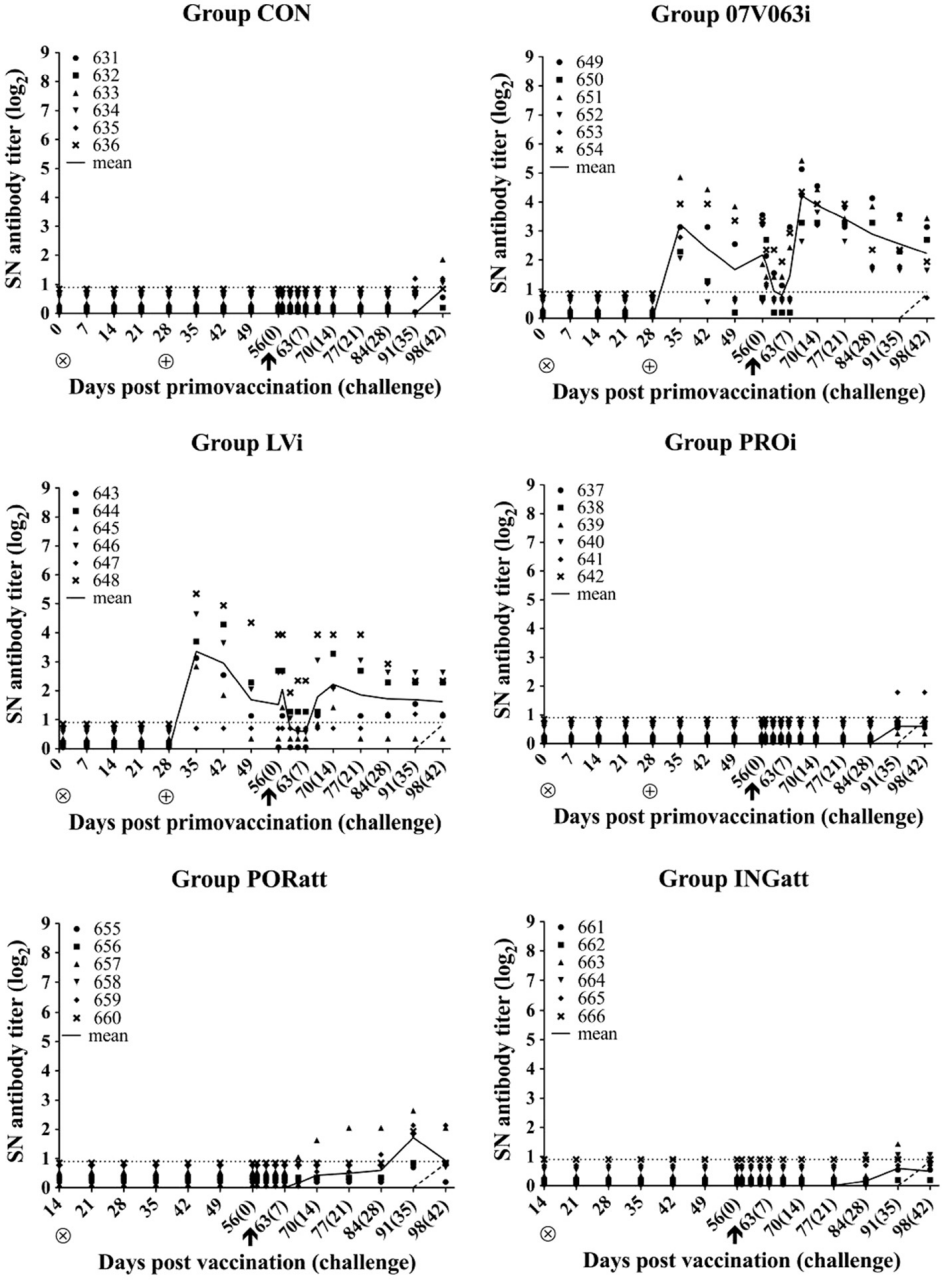
**07 V063-neutralizing antibody titers (log**_**2**_**) after vaccination and 07 V063 challenge for group CON (Mock-vaccinated control), 07 V063i (BEI-inactivated 07 V063), LVi (BEI-inactivated LV), PROi (Progressis®), PORatt (Porcilis® PRRS) and INGatt (Ingelvac® PRRS MLV)**. ⊗ = primo vaccination; ⊕ = booster vaccination; ↑ = challenge. Symbols represent individual animals and solid lines represent mean SN titers calculated on all animals present in each group. The dashed line indicates the mean titers for all animals in group CON. The dotted line marks the detection limit for the SN test.

In group CON, VN antibodies were not detected until 5 weeks post challenge. Even at 6 weeks post challenge, not all the animals from this group were positive for VN antibodies (Figure 3, CON). In group 07 V063i, 07 V063-specific VN antibodies were already detected upon booster vaccination. The mean VN antibody titer decreased immediately post challenge, but increased again 10 days after infection. Some animals had no or undetectable VN antibodies in the period between 2 weeks before and 10 dpc, but after this period, VN antibodies were detected in all animals. The mean VN antibody titer was significantly higher compared to group CON in the period between 1 week after booster vaccination and 5 weeks post challenge, reaching mean values ranging from 1.1 to 4.2 log_2_ (p < 0.05) (Figure 3, 07 V063i). A similar pattern was observed in group LVi: 07 V063-neutralizing antibodies were already detected at 1 week after booster vaccination. VN antibody titers initially decreased post challenge and increased again from 10 dpc. Some animals turned negative for VN antibodies in the period between 1 week before and 10 days after the challenge, but after this period, VN antibodies were detected in all 6 animals. Remarkably, one animal in this group did not show neutralizing antibodies earlier than 4 weeks post challenge. The VN antibody titers were significantly higher compared to the control group at 1 and 2 weeks after booster vaccination and at 10 and 14 dpc, reaching mean values of 3.5, 3.1, 1.9 and 2.3 log_2_, respectively (p < 0.05) (Figure 3, LVi). Pigs that were vaccinated with Progressis® (two shots), Porcilis® PRRS (single shot) and Ingelvac® PRRS (single shot) showed a roughly similar VN antibody response as the animals in the control group. In the Porcilis® vaccinated group, a slight increase of VN antibodies was noticed at 5 weeks post challenge, but there were no significant differences with group CON (Figure 3, PORatt). In summary, both BEI-inactivated vaccines induced a strong 07 V063-specific VN antibody response after booster vaccination, while the commercial vaccines, both inactivated and attenuated (EU or NA genotype), did not induce a VN antibody response against 07 V063.

### Vaccination experiment with PRRSV isolate 08 V194

#### Clinical examination

All animals remained in good health after they were vaccinated. No local or systemic vaccine side effects were noted throughout the trial period. One pig of group PORatt2 died at day 84 at the moment of blood collection. The daily rectal temperatures varied in all groups and no statistically significant differences were observed. Challenge with PRRSV isolate 08 V194 induced moderate fever (higher than 39.5°C, but not higher than 40.4°C) within 7 days post infection in 24 out of 31 inoculated pigs. The 7 remaining animals did not develop fever. By 8 days post challenge, fever had disappeared in all animals.

#### Viremia

**Figure 4:**
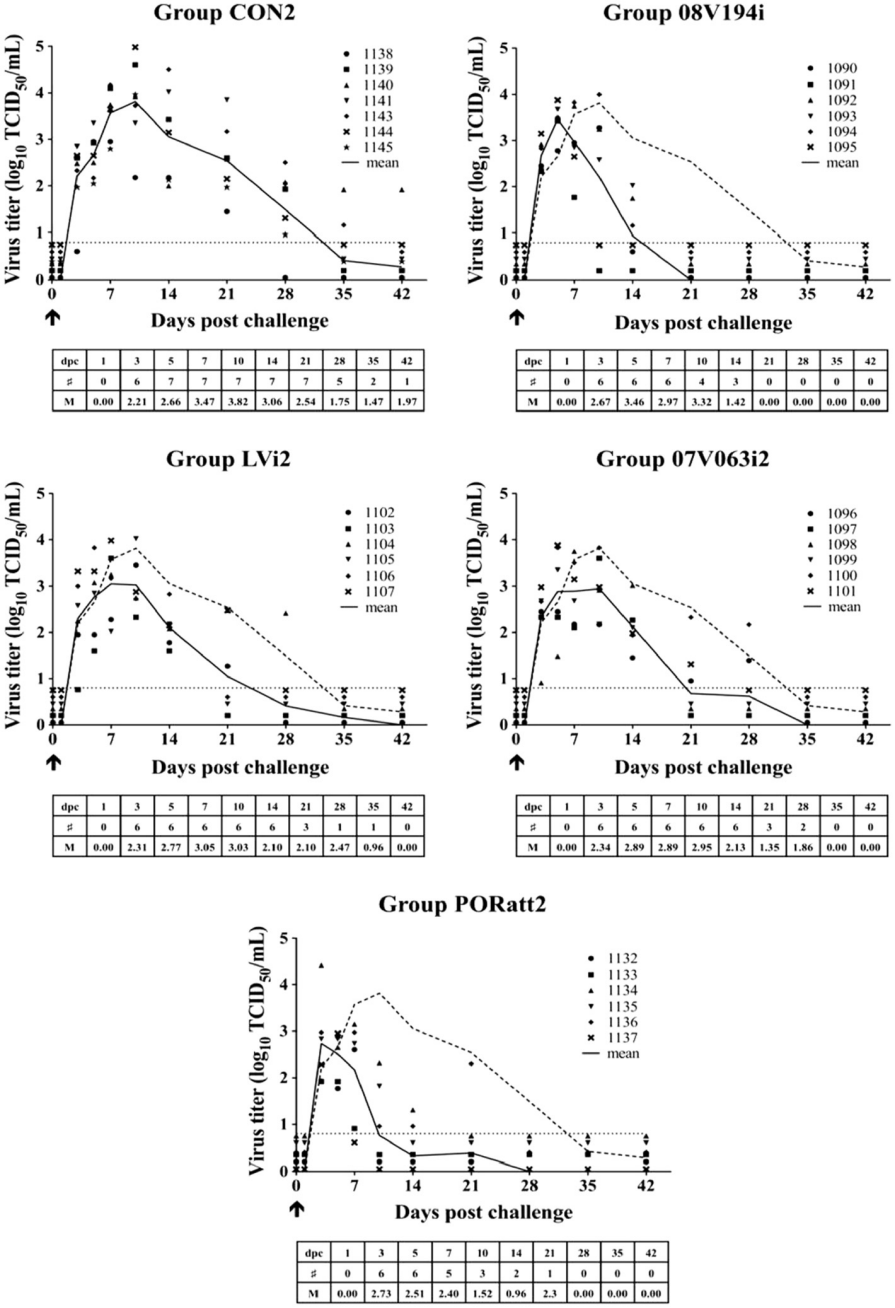
**Serum-virus titers after 08 V194 challenge for group CON2 (Mock-vaccinated control), 08 V194i (BEI-inactivated 08 V194), LVi2 (BEI-inactivated LV), 07 V063i2 (BEI-inactivated 07 V063) and PORatt2 (Porcilis® PRRS).** Virus titers in serum (log_10_ TCID_50_/mL) were determined by virus titration on PAM, followed by immunoperoxidase staining for the PRRSV nucleocapsid protein. ↑ = challenge. Symbols represent individual animals and solid lines represent mean virus titers calculated on all animals present in each group. The dashed line indicates the mean titers for all animals in group CON2. The dotted line marks the detection limit for virus titration. Mentioned in the table: # = the number of viremic animals in the different groups at different time points. M = mean virus titer of all viremic animals in the group at different time points.

Upon challenge, all animals became viremic. In the control group (group CON2), a maximum mean virus titer of 3.8 log_10_ TCID_50_/mL was reached at 10 dpc. Subsequently, a decline in virus titer was observed and virus was no longer detectable in the serum at 4, 5 or 6 weeks after challenge, depending on the animal. Still, 1 piglet remained virus positive till 6 weeks post challenge (Figure 4, CON2). In the BEI-inactivated 08 V194 vaccinated group (group 08 V194i), the viremic peak at day 5 was not reduced compared to group CON2, but the mean virus titer at day 14 was significantly reduced (p < 0.05) and from 21 dpc, virus could no longer be detected in any of the piglets (Figure 4, 08 V194i). The number of viremic piglets in group 08 V194i was significantly lower compared to group CON2 on day 21 and 28 post challenge (p < 0.05). Mean virus titers in the group vaccinated with BEI-inactivated LV virus (group LVi2) were comparable to those in group CON2, reaching 3.0 log_10_ TCID_50_/mL at 10 dpc, and no significant differences could be detected at any time-point between group LVi2 and group CON2. For 3 animals of this group, virus was cleared from the blood at 3 weeks, for 2 others at 4 weeks and in the remaining animal at 5 weeks post challenge (Figure 4, LVi2). In the BEI-inactivated 07 V063 vaccinated group (group 07 V063i2), viremia was detected in all animals, with a peak around 5–10 dpc. The viremic phase showed a similar pattern as for group LVi2 and viremia was also not significantly reduced compared to group CON2. Viremia disappeared in all animals by 5 weeks after challenge (Figure 4, 07 V063i2). The mean virus titer in the group vaccinated with Porcilis® PRRS (group PORatt2) reached 2.7 log_10_ TCID_50_/mL at 3 days and 2.5 log_10_ TCID_50_/mL at 5 dpc, but virus titers were not significantly different from those in group CON2 at these time points. At later time points however, virus titers were significantly reduced compared to group CON2 (p < 0.05). Moreover, viremia in group PORatt2 was already cleared at 10 dpc for 3 animals and at 28 dpc, all animals were negative (Figure 4, PORatt2). From 14 till 28 dpc, the total number of viremic animals in group PORatt2 was significantly lower than in group CON2 (p < 0.05). In summary, groups 08 V194i and PORatt2 showed a significantly shortened viremia and a significant decrease in the number of viremic piglets compared to the mock-vaccinated control group, while no such effect was seen in groups LVi2 and 07 V063i2.

#### 08 V194-specific antibodies

**Figure 5:**
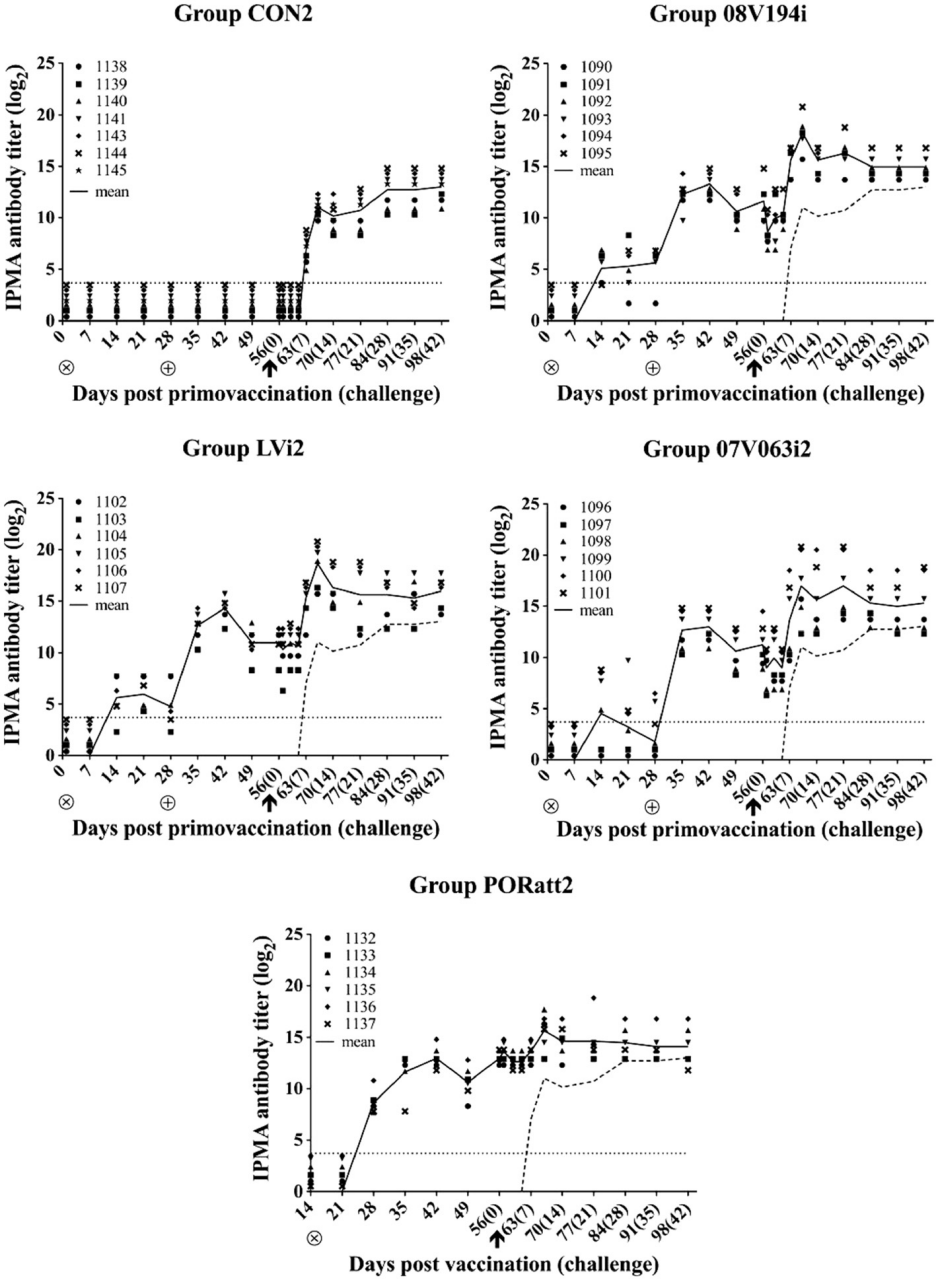
**08 V194-specific IPMA antibody titers (log**_**2**_**) after vaccination and 08 V194 challenge for group CON2 (Mock-vaccinated control), 08 V194i (BEI-inactivated 08 V194), LVi2 (BEI-inactivated LV), 07 V063i2 (BEI-inactivated 07 V063) and PORatt2 (Porcilis® PRRS).** ⊗ = primo vaccination; ⊕ = booster vaccination; ↑ = challenge. Symbols represent individual animals and solid lines represent mean IPMA titers calculated on all animals present in each group. The dashed line indicates the mean titers for all animals in group CON2. The dotted line marks the detection limit for the IPMA test.

All CON2 animals had virus-specific serum antibodies starting from 7 dpc (Figure 5, CON2). All 6 animals of group 08 V194i seroconverted at 2 or 3 weeks after the first vaccination. Similarly, all animals of group LVi2 showed virus-specific antibodies 2 weeks after the first vaccination. In group 07 V063i2, 08 V194-specific antibodies were detected from 2 weeks after primo vaccination and all animals seroconverted after booster vaccination. Antibody titers in all 3 vaccinated groups were significantly higher compared to group CON2 from 1 week after booster vaccination up till 21 dpc (p < 0.05) (Figure 5, 08 V194i, LVi2 and 07 V063i2). After 21 dpc, mean antibody titers in groups 08 V194i, LVi2 and 07 V063i2 remained higher compared to the control group, although differences were not significant. In group PORatt2, all pigs showed positive antibody titers at 2 weeks after vaccination; the antibody titers were significantly higher compared to group CON2 starting from 2 weeks after vaccination up till 3 weeks post challenge (p < 0.05) (Figure 5, PORatt2). In summary, the course of the IPMA antibody titers in all groups were similar to those described in previous studies and the former experiment in this study [24,25]. 

#### 08 V194-specific virus-neutralizing antibodies

**Figure 6:**
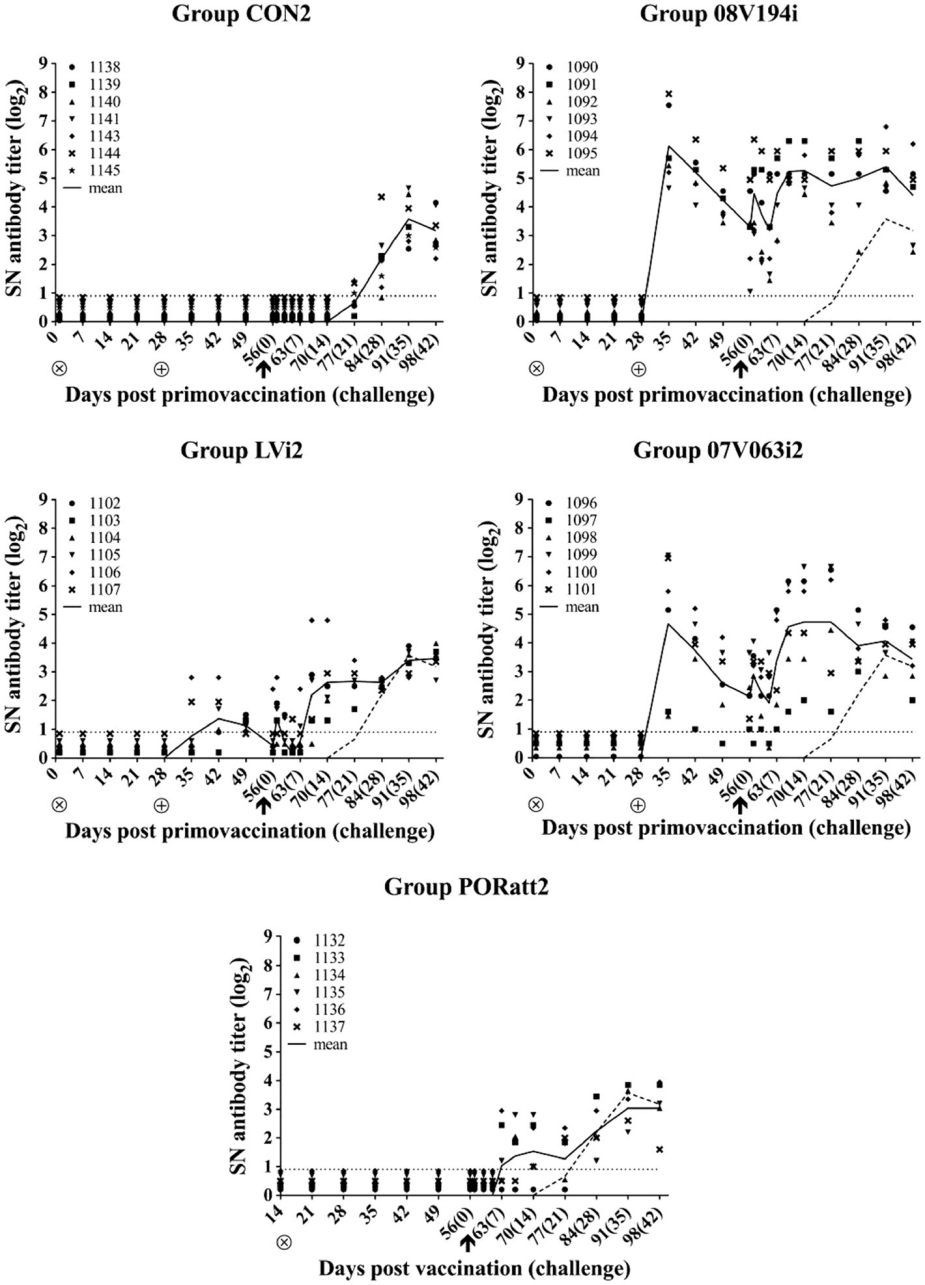
**08 V194-neutralizing antibody titers (log**_**2**_**) after vaccination and 08 V194 challenge for group CON2 (Mock-vaccinated control), 08 V194i (BEI-inactivated 08 V194), LVi2 (BEI-inactivated LV), 07 V063i2 (BEI-inactivated 07 V063) and PORatt2 (Porcilis® PRRS).** ⊗ = primo vaccination; ⊕ = booster vaccination; ↑ = challenge. Symbols represent individual animals and solid lines represent mean SN titers calculated on all animals present in each group. The dashed line indicates the mean titers for all animals in group CON2. The dotted line marks the detection limit for the SN test.

Starting from 21 dpc, 3 pigs of group CON2 showed a VN antibody titer and by 35 dpc, VN antibodies had appeared in all mock-vaccinated pigs (Figure 6, CON2). All six pigs of group 08 V194i showed high VN antibody titers at 1 week after the booster vaccination and this remained so until the end of the experiment (Figure 6, 08 V194i). VN antibody titers were significantly higher in group 08 V194i compared to group CON2 from 1 week after booster vaccination until 5 weeks post challenge, with mean values ranging from 3.2 to 6.2 log_2_ (p < 0.05). All animals of group LVi2 seroconverted for VN antibodies at least once within 3 weeks after booster vaccination, but VN antibody titers remained low and were only significantly higher than group CON2 at 14 dpc, reaching a mean value of 2.6 log_2_ (p < 0.05) (Figure 6, LVi2). A similar pattern as in group 08 V194i was observed in group 07 V063i2, where 08 V194-neutralizing antibodies could already be detected at 1 week after booster vaccination. Two animals turned negative for VN antibodies in the period between 2 weeks post booster vaccination and 10 dpc, but after this period, VN antibodies were consistently detected in all 6 animals. The mean VN antibody titer in group 07 V063i2 was significantly higher compared to group CON2 in the period between 1 week after booster vaccination and 4 weeks post challenge (except for time-point 5 dpc), reaching mean values ranging from 2.1 to 4.7 log_2_ (p < 0.05) (Figure 6, 07 V063i2). None of the animals in group PORatt2 showed 08 V194-specific VN antibodies before challenge, but VN antibodies already appeared between 5 and 10 dpc. The mean VN antibody titer in group PORatt2 was slightly but not significantly higher compared to group CON2 between 7 and 14 dpc, reaching a maximum of 1.6 log_2_ at 14 dpc (Figure 6, PORatt2). In summary, both BEI-inactivated 08 V194 (homologous) and 07 V063 (heterologous) vaccines induced a strong 08 V194-specific VN antibody response upon booster vaccination, while this was not the case for the heterologous BEI-inactivated LV vaccine and the commercial attenuated vaccine.

## Discussion

PRRSV causes severe reproductive disorders in sows and boars and is associated with the porcine respiratory disease complex. The virus is difficult to control and has become endemic in many major swine-producing countries, leading to tremendous economic losses worldwide [8]. To control the disease, several commercial attenuated and inactivated vaccines are currently available. However, when used in the field, these vaccines have met with variable degrees of success. Reported outbreaks of clinical PRRS in vaccinated pigs have led to doubts about the efficacy of currently available vaccines [26]. New vaccination strategies are needed to achieve the goals of local and regional elimination of PRRSV and it is generally accepted that a continuous update of vaccine strains is necessary to reach an acceptable level of protection in the field, even within geographical areas of limited size. A recent study by Vanhee et al. (2009) [25] showed that a PRRSV LV-based BEI-inactivated vaccine induces LV-specific VN antibodies in PRRSV-negative animals and offers partial protection upon homologous challenge. In that study, it was however not assessed if such a vaccine can be adapted to field variants of PRRSV that are genetically and antigenically divergent from the currently used vaccine strains. The main objective of the current study was to assess the efficacy of experimental BEI-inactivated vaccines, based on recent PRRSV field isolates (07 V063 and 08 V194), against homologous and heterologous challenge. A commercial inactivated (Progressis®) and two commercial attenuated (Porcilis® PRRS and Ingelvac® PRRS MLV) PRRSV vaccines were included in the study and served as a reference. Vaccine efficacy was assessed by evaluating the viremia upon challenge – a factor directly linked with viral pathogenesis and spread.

The 07 V063- and 08 V194-based inactivated PRRSV vaccines were effective in partially protecting naïve pigs upon homologous challenge. They shortened viremia with 2 (07 V063) and 3 (08 V194) weeks compared to the viremic phase in the respective mock-vaccinated groups, which lasted roughly 1 month. BEI-inactivated LV vaccines were included to assess the impact of strain variability on vaccine efficacy. We found no reduction in 07 V063 viremia after the use of an inactivated LV-based vaccine and only a non-significant reduction of viremia upon challenge with 08 V194. Similarly, a 07 V063-based BEI-inactivated PRRSV vaccine did not significantly reduce viremia upon challenge with the 08 V194 isolate. The Progressis® vaccine did not provide any virological protection, since viremia was observed for 4 weeks upon challenge with the 07 V063 isolate. This is in line with the results from previous studies, showing that the commercial inactivated vaccines appear not to influence viremia, even in nearly homologous conditions [21,24,27]. Vaccination with the EU-genotype attenuated vaccine reduced the duration of viremia upon challenge with 07 V063 with approximately one week. In animals challenged with 08 V194, this vaccine shortened viremia from 5 to 2 weeks. The NA-genotype attenuated vaccine reduced viremia in 07 V063-challenged animals with approximately one week. Hence, despite the concerns regarding the efficacy of the attenuated vaccine used on both farms, the results of our study indicate that the use of this vaccine in PRRS-naïve pigs can clearly limit viremia. These results are in line with earlier studies published by Cano et al. (2007) [28] and Scortti et al. (2006) [29], showing that attenuated vaccines can be successful in controlling and reducing clinical disease upon homologous and heterologous challenge.

In the field, PRRSV vaccination is mainly performed in sows. Therefore, we reasoned it would also be interesting to assess the antibody response induced by the vaccines, since maternal antibodies play a pivotal role in the passive (colostral) immunity that protects piglets during their first weeks of life [30]. Although resolution of PRRSV infection is not always directly correlated with the neutralizing antibody response [31], there is ample evidence that neutralizing antibodies can facilitate virus clearance and, when present in sufficient amounts, may even provide a sterilizing immunity [32-35]. IPMA and SN tests were performed to evaluate the capacity of the vaccines to induce or prime a challenge virus-specific (neutralizing) antibody response.

Vaccination with BEI-inactivated 07 V063 or 08 V194 vaccines consistently induced sizable titers of homologous PRRSV-neutralizing antibodies after at least two immunizations given four weeks apart. Interestingly, vaccination with BEI-inactivated LV also induced sizeable titers of 07 V063-neutralizing antibodies. Similarly, both 07 V063- and LV-based vaccines induced 08 V194-neutralizing antibodies, with the LV-induced titers being lower than the 07 V063-induced titers. In all groups vaccinated with a BEI-inactivated vaccine, the VN titers dropped immediately after challenge, which may indicate that the antibodies were consumed during their interaction with virus early in infection. However, after this initial drop in VN antibody titers, VN antibodies quickly reappeared. The fast appearance of VN antibodies upon challenge is in agreement with the findings in the study of Vanhee et al. (2009) [25] and demonstrates the potential of priming the neutralizing antibody response by immunization with a high dose of inactivated PRRSV. Although it has been reported that the PRRSV-specific neutralizing antibody response is to a large extent strain-specific and a lack in cross-neutralization may occur even between genetically closely related virus strains [36,37], our data show that cross-neutralization between genetically different isolates can occur. In the animals vaccinated with the commercial inactivated PRRSV vaccine Progressis® (first experiment), neither the IPMA nor the VN antibody response was influenced before or after challenge with 07 V063. This is in line with the studies by Zuckermann et al. (2007) [24] and Vanhee et al. (2009) [25], where they used the same vaccine and the LV strain as challenge virus: no clear induction of challenge virus-specific (neutralizing) antibodies was observed upon vaccination with the commercial inactivated PRRSV vaccine and only a moderate anamnestic antibody response was observed upon challenge of the vaccinated animals. The apparent limited immunogenicity of this vaccine may relate to the inactivation procedure used, strain variability, antigenic dose, adjuvant, … Further research is necessary to elucidate this. In the animals vaccinated with the commercial attenuated vaccines, either based on EU- (Porcilis® PRRS) or NA- (Ingelvac® PRRS MLV) type virus, a low or non-detectable VN antibody response was observed, which is in agreement with the results of Lopez et al. (2004) [33]. None of the attenuated vaccines were able to induce a faster neutralizing antibody response upon challenge. The data obtained in this study have provided the basis for an ongoing field study on the effect of different vaccines at the farm level, more specifically on the effects of vaccination of sows on the passive immunity transferred to piglets.

In the 07 V063- and 08 V194-challenged groups vaccinated with a BEI-inactivated vaccine homologous to the challenge virus, a correlation was seen between the induction of virus-specific neutralizing antibodies and reduction in viremia, indicating that VN antibodies may contribute to protection against the virus. However, the induction of homologous VN antibodies was not sufficient to completely protect the animals, as it still permitted the development of a viremia post-challenge that lasted at least one week. Possibly, higher VN antibody titers are needed at the time of challenge to offer full protection against the high dose of virus used to infect the animals. Administration of a heterologous BEI-inactivated vaccine was not sufficient to significantly reduce viremia in the animals upon challenge. Since the BEI-inactivated vaccines used in this study induced antibodies that could neutralize the homologous as well as the heterologous challenge virus in *in vitro* seroneutralization assays, it was somewhat surprising that these vaccines could only limit viremia under the homologous challenge conditions, and not when the heterologous challenge virus was used. The exact reason behind this remains currently unknown, but several possible explanations suggest themselves. For instance, it is possible that induction of virus-specific neutralizing antibodies is not sufficient and that BEI-inactivated PRRSV vaccines must promote other immune mechanisms (e.g. via cross-presentation to T-cells) to provide a significant degree of protection upon challenge. On the other hand, it can be speculated that, although the vaccine-induced antibodies can bind and neutralize the homologous and heterologous challenge virus to a similar extent in *in vitro* SN assays, they recognize the homologous virus with a higher affinity. Affinity differences may explain a reduced binding and neutralization of heterologous virus *in vivo*, as the binding conditions for (VN) antibodies are likely more stringent *in vivo* than in the *in vitro* SN assays. Under homologous challenge conditions, the antibodies have undergone optimal challenge virus-specific affinity maturation, while this is not the case under heterologous challenge conditions. In theory, the presence of vaccine-induced antibodies that cross-react with a heterologous challenge virus may even prevent the selection of high-affinity (VN) antibodies against this challenge virus (original antigenic sin). Clearly, this matter requires further investigation in the future. Despite the absence of a clear challenge virus-specific VN antibody response, the commercial attenuated vaccines do provide a partial virological protection, roughly similar to the protection provided by the autogenous BEI-inactivated vaccines. This observation points towards a significant role of other attenuated vaccine-induced immune mechanisms (e.g. cell-mediated immunity) in the protection against PRRSV infection [24,31,38]. Vaccination with the commercial inactivated vaccine Progressis® did not induce VN antibodies and neither did it provide any degree of protection upon challenge.

Considering the similar efficacy of the attenuated vaccines against both challenge isolates used in this study, it can be questioned whether the use of autogenous inactivated vaccines is advantageous over the use of the current attenuated vaccines. However, while the efficacy of the attenuated vaccines against new virus variants can be unpredictable, our data demonstrate that an (adaptable) autogenous BEI-inactivated vaccine can provide a more or less standardized, predictable degree of protection against a specific virus variant, which may prove useful in case virus variants emerge that escape the immunity induced by the attenuated vaccines. In the near future, additional research will be conducted to further substantiate this. Also, although the production of autogenous inactivated vaccines as described in this study may appear too elaborate and costly (virus isolation, adaptation to cell culture, high dose needed,…), further optimization of the production process should make future use of these vaccines more feasible.

## Conclusions

The current study assessed the protective capacity of different experimental and commercial vaccines against challenge with two recent PRRSV field isolates. Experimental BEI-inactivated vaccines based on these field isolates significantly shortened viremia upon homologous challenge. Despite the concerns regarding the efficacy of the commercial attenuated vaccines used on the farms where the field isolates were obtained, use of commercial attenuated vaccines resulted in a similar reduction of the viremic phase. In contrast, the experimental BEI-inactivated vaccines did not significantly reduce viremia upon heterologous challenge and the commercial inactivated vaccine had no apparent effect.

While the BEI-inactivated vaccines (both homologous and heterologous) induced challenge virus-specific neutralizing antibodies, this was not the case for the commercial inactivated and attenuated vaccines. The results illustrate that the capacity of a vaccine to induce challenge virus-specific neutralizing antibodies does not necessarily correlate with protection against the challenge virus and vice versa, suggesting that not only vaccine-induced antibodies, but also other vaccine-induced immune mechanisms can contribute to PRRSV-specific protective immunity.

The observation that homologous BEI-inactivated vaccines can provide a more or less standardized, predictable degree of protection against a specific virus variant suggests that such vaccines may prove useful in case virus variants emerge that escape the immunity induced by the attenuated vaccines. Future research will allow optimization and simplification of the production process of the adaptable BEI-inactivated vaccines and give further insights into the mechanisms of protection induced by these vaccines.

## Methods

### Cells and viruses

Porcine alveolar macrophages (PAMs) were derived from 3-week-old (just weaned) piglets, purchased from a PRRSV- and *Mycoplasma Hyopneumoniae*-negative farm. After isolation, the morphology of PAMs was checked visually via light microscopy. No specific tests were performed to detect PCV2. PAMs and MARC-145 cells were cultivated as described before [39].

The Belgian PRRSV isolates used in this study originated from two farms showing clinical signs compatible with PRRS in sows or growing pigs. The two isolates were randomly selected from 19 isolates obtained between 2007 and 2010. At the moment of sampling, sows of both herds were vaccinated with a EU-genotype attenuated vaccine (Porcilis® PRRS). PRRSV isolate 07 V063 was isolated from fetal tissue by inoculating tissue suspensions on PAM. This isolate has been used in recent studies by Karniychuk et al. (2011; 2012) [40,41], describing viral, clinical and pathological data. Similarly, the 08 V194 isolate was obtained by inoculating the serum of 14-week-old piglets on PAM. Both isolates were also adapted to MARC-145 cells by repeated passages. For challenge, macrophage-grown stocks were prepared of the isolates 07 V063 (2^nd^ passage on PAM) and 08 V194 (5^th^ passage on PAM).

For vaccine preparation, MARC-145 cell culture supernatants of 07 V063 (2^nd^ passage on PAM + 2 passages on MARC-145), 08 V194 (2^nd^ passage on PAM + 4 passages on MARC-145) and LV (2^nd^ passage on PAM + 5 passages on MARC-145), were purified via ultracentrifugation as previously described by Vanhee et al. (2009) [25].

### Genome sequencing and phylogenetic analysis

To determine if adaptation to the MARC-145 cell line resulted in mutations in the structural ORFs, ORF2-7 of MARC-145-grown 07 V063, 08 V194 and LV were sequenced and compared with those of original macrophage-grown 07 V063, 08 V194 and LV. Sequencing was performed as described before [42]. Nucleotide sequences were submitted to Genbank under accession numbers [GenBank: GU737264 (07 V063) and [GenBank: GU737265 (08 V194).

Amino acid (aa) sequences were subsequently derived and analysed using CLC Free workbench 4. The aa sequences of all structural proteins of MARC-145-grown 07 V063, 08 V194 and LV were 100% identical to those of the corresponding proteins of original macrophage-grown virus. The clear difference in aa sequence between both 07 V063 and 08 V194 and the Porcilis® PRRS strain allowed their classification as EU wild-type viruses that are not of vaccine origin.

### Virus inactivation and quality control

Purified virus (07 V063, 08 V194 and LV) was suspended in RPMI 1640 (Invitrogen) to a titer of 10^8^ TCID_50_/mL. Subsequently, the virus was inactivated using BEI as described before [25], and inactivated virus was stored at −70°C. To confirm that all virus was completely inactivated, a complete vaccine dose of 07 V063, 08 V194 and LV was inoculated on MARC-145 cells and subsequently passaged twice. As a positive control, MARC-145 cells were inoculated with 1 mL of non-inactivated 07 V063, 08 V194 or LV. The MARC-145 cells were routinely checked for cytopathic effect (CPE) and ultimately stained for the PRRSV nucleocapsid protein via an immunoperoxidase staining using monoclonal antibody 13E2 [43]. No CPE or positive nucleocapsid staining was detected in cells that were inoculated with inactivated virus, while clear CPE and nucleocapsid staining were observed in cell cultures that were inoculated with non-inactivated virus.

Since conservation of entry of inactivated virus may serve as a quality control for the preservation of antigenic properties, the effect of BEI inactivation on virus attachment and internalization into macrophages was examined as described previously [25,44]. Non-inactivated virus suspensions were included as positive controls. The entry experiment showed that the binding and internalization kinetics of all BEI-inactivated virus stocks are similar to those observed for the non-inactivated virus stocks.

### Pigs and experimental design

Sixty-seven four-week-old piglets were purchased from a PRRSV-negative farm and their PRRSV-seronegative status was confirmed by IPMA upon arrival. The animals were housed in isolation units with HEPA-filtered air and kept during 7 days to allow adaptation to the new conditions. Two experiments were performed (Table 1). All animal experiments were approved by the local ethical committee of the Faculty of Veterinary Medicine, Ghent University.

**Table 1:** Experimental design of vaccination-challenge experiments

**Group**	**Vaccination**	**Age in weeks**	**Challenge strain (13 weeks)**
Experiment 1 (n)			
CON (6)	Mock	5 and 9	07 V063
07 V063i (6)	BEI-inactivated 07 V063	5 and 9	07 V063
LVi (6)	BEI-inactivated LV	5 and 9	07 V063
PROi (6)	Progressis®	5 and 9	07 V063
PORatt (6)	Porcilis® PRRS	7	07 V063
INGatt (6)	Ingelvac® PRRS	7	07 V063
Experiment 2 (n)			
CON2 (7)	Mock	5 and 9	08 V194
08 V194i (6)	BEI-inactivated 08 V194	5 and 9	08 V194
LVi2 (6)	BEI-inactivated LV	5 and 9	08 V194
07 V063i2 (6)	BEI-inactivated 07 V063	5 and 9	08 V194
PORatt2 (6)	Porcilis® PRRS	7	08 V194

#### Vaccination experiment with PRRSV isolate 07 V063

Thirty-six pigs were randomly divided into six groups. An oil-in-water (o/w) diluent, normally used in the commercial pseudorabies virus vaccine Suvaxyn Aujeszky (Fort Dodge Animal Health), was used as an adjuvant and is further referred to as o/w Suvaxyn. A first group (group CON, *n* = 6 pigs) served as a mock-vaccinated control group and received 1 mL RPMI 1640 in 1 mL o/w Suvaxyn intramuscularly at 5 and 9 weeks of age. Three other groups were vaccinated twice intramuscularly at 5 (primo vaccination) and 9 (booster vaccination) weeks of age. Group 07 V063i (*n* = 6 pigs) was vaccinated with 1 mL BEI-inactivated MARC-145-grown 07 V063 (10^8^ TCID_50_) in 1 mL o/w Suvaxyn and group LVi (*n* = 6 pigs) was vaccinated with 1 mL BEI-inactivated MARC-145-grown LV (10^8^ TCID_50_) in 1 mL o/w Suvaxyn. Group PROi (*n* = 6 pigs) received 2 mL of a commercial European type inactivated PRRSV vaccine (Progressis®, Merial, strain P120: min 2,5 log IF Units). Groups PORatt (*n* = 6 pigs) and INGatt (*n* = 6 pigs) were vaccinated once intramuscularly with the European type attenuated vaccine (Porcilis® PRRS, Intervet, 10^4^ TCID_50_/2 mL) and the American type attenuated vaccine (Ingelvac® PRRS MLV, Boehringer Ingelheim, 10^4.9^ TCID_50_/2 mL), respectively, at the age of 7 weeks. At 13 weeks of age, all pigs were challenged intranasally with PRRSV 07 V063 (10^6^ TCID_50_) in phosphate buffered saline (PBS) (2,5 ml per nostril). Blood samples were taken by jugular venipuncture weekly after (primo) vaccination and at 0, 1, 3, 5, 7, 10, 14, 21, 28, 35 and 42 dpc. Serum was collected and stored at −70°C. Serum samples for IPMA and VN antibody detection were incubated for 30 min at 56°C prior to freezing.

#### Vaccination experiment with PRRSV isolate 08 V194

In a second experiment, 31 piglets were randomly assigned to five treatment groups. Group CON2 (*n* = 7 pigs) served as a mock-vaccinated control group and received 1 mL RPMI 1640 in 1 mL o/w Suvaxyn intramuscularly at 5 and 9 weeks of age. Three other groups were vaccinated twice intramuscularly at 5 (primo vaccination) and 9 (booster vaccination) weeks of age. Group 08 V194i (*n* = 6 pigs) was vaccinated with 1 mL BEI-inactivated MARC-145-grown 08 V194 (10^8^ TCID_50_) in 1 mL o/w Suvaxyn, group LVi2 (*n* = 6 pigs) was vaccinated with 1 mL BEI-inactivated MARC-145-grown LV (10^8^ TCID_50_) in 1 mL o/w Suvaxyn and group 07 V063i2 (*n* = 6 pigs) was vaccinated with 1 mL BEI-inactivated MARC-145-grown 07 V063 (10^8^ TCID_50_) in 1 mL o/w Suvaxyn. At 7 weeks of age, pigs of group PORatt2 (*n* = 6 pigs) were vaccinated intramuscularly with Porcilis® PRRS at a dose of 10^4^ TCID_50_ per pig. PRRSV-isolate 08 V194 at a dose of 10^6^ TCID_50_ was used to inoculate all pigs intranasally (2,5 ml per nostril) at the age of 13 weeks. The same experimental design was used as in the first experiment.

### Virus titration and serological examinations

Virus titers in serum were determined by virus titration on PAM following a standard procedure [32]. 24-h cultivated PAM were inoculated with 10-fold dilution series of the serum samples. 72 hours post inoculation, cells were fixed and an immunoperoxidase staining with monoclonal antibody 13E2 against the PRRSV nucleocapsid protein was performed to visualize infection in the cells [43]. The titers were calculated as described by Reed and Muench (1938) [45] and expressed as TCID_50_/mL. To check the sensitivity of the PAM, all cell batches were assayed in virus titrations using a PRRSV stock (LV) with a known virus titer.

Serum samples were examined for the presence of PRRSV-specific antibodies using an IPMA as described by Labarque et al. (2000) [32]. To detect antibodies against 07 V063 (1^st^ experiment), an IPMA was performed on 07 V063-infected MARC-145 cells. To detect antibodies against 08 V194 (2^nd^ experiment), an IPMA was performed on 08 V194-infected MARC-145 cells. VN antibodies were detected by seroneutralization assays on MARC-145 cells using the respective PRRSV challenge isolate. Each serum sample was tested in duplicate. Briefly, serum samples were twofold serially diluted and an equal volume of a PRRSV 07 V063 (2^nd^ passage on PAM + 2 passages on MARC-145) or 08 V194 (2^nd^ passage on PAM + 4 passages on MARC-145) suspension (titer 2 × 10^3^ TCID_50_/mL) was added to each dilution. After mixing, the plates were incubated at 37°C for 1 h and 50 μl of the mixture was subsequently transferred to confluent monolayers of MARC-145 cells in 96-well plates. Cells were screened for 7 days after inoculation and the neutralization titer of the sera was recorded as the reciprocal of the highest dilution that inhibited CPE in 50% of the inoculated wells. To check the sensitivity to PRRSV infection of different passages of MARC-145 cells, control titrations using PRRSV stocks (isolate 07 V063 and isolate 08 V194) with a known virus titer were performed in parallel with each neutralization assay.

### Statistical analysis

Antibody titers and virus titers were analyzed by Kruskall-Wallis test, followed by Dunn’s multiple comparisons test to determine significant differences with the control groups at different time points. Samples, that tested negative in IPMA, VN or virus isolation were consequently given a numerical value of 0.0. A two-tailed Fisher’s exact test was used to determine significant differences between the number of viremic animals in the vaccinated groups and the control groups at different time points. An overall *p* value of 0.05 was taken as the level of statistical significance. All statistical analyses were performed using GraphPad Prism version 5.0a (GraphPad Software, San Diego, California, USA).

## Competing interests

The authors declare that they have no competing interests.

## Authors’ contributions

MFG carried out the isolation of both PRRSV isolates, performed the vaccination studies and statistical analyses. He wrote the manuscript and participated in the study design. MV participated in both vaccination studies and helped writing the manuscript. WVB participated in the second experiment and helped to write the manuscript. JVD carried out sequencing and phylogenetic analyses. UUK participated in the first experiment. HJN conceived the study, coordinated the work and helped in writing the manuscript. All authors read and approved the final manuscript.
